# Supplementary material for: Rho-Kinase Inhibitors for the Treatment of Refractory Diabetic Macular Oedema
Source: Cells. 2021 Jul 3;10(7):1683. doi: 10.3390/cells10071683 (PMC8307715; doi:10.3390/cells10071683)
Supplement: Supplementary file 1 [file cells-10-01683-s001.zip › Flow-diagram_PRISMA2020.pdf]

PRISMA 2020 flow diagram for new systematic reviews which included searches of databases and registers only

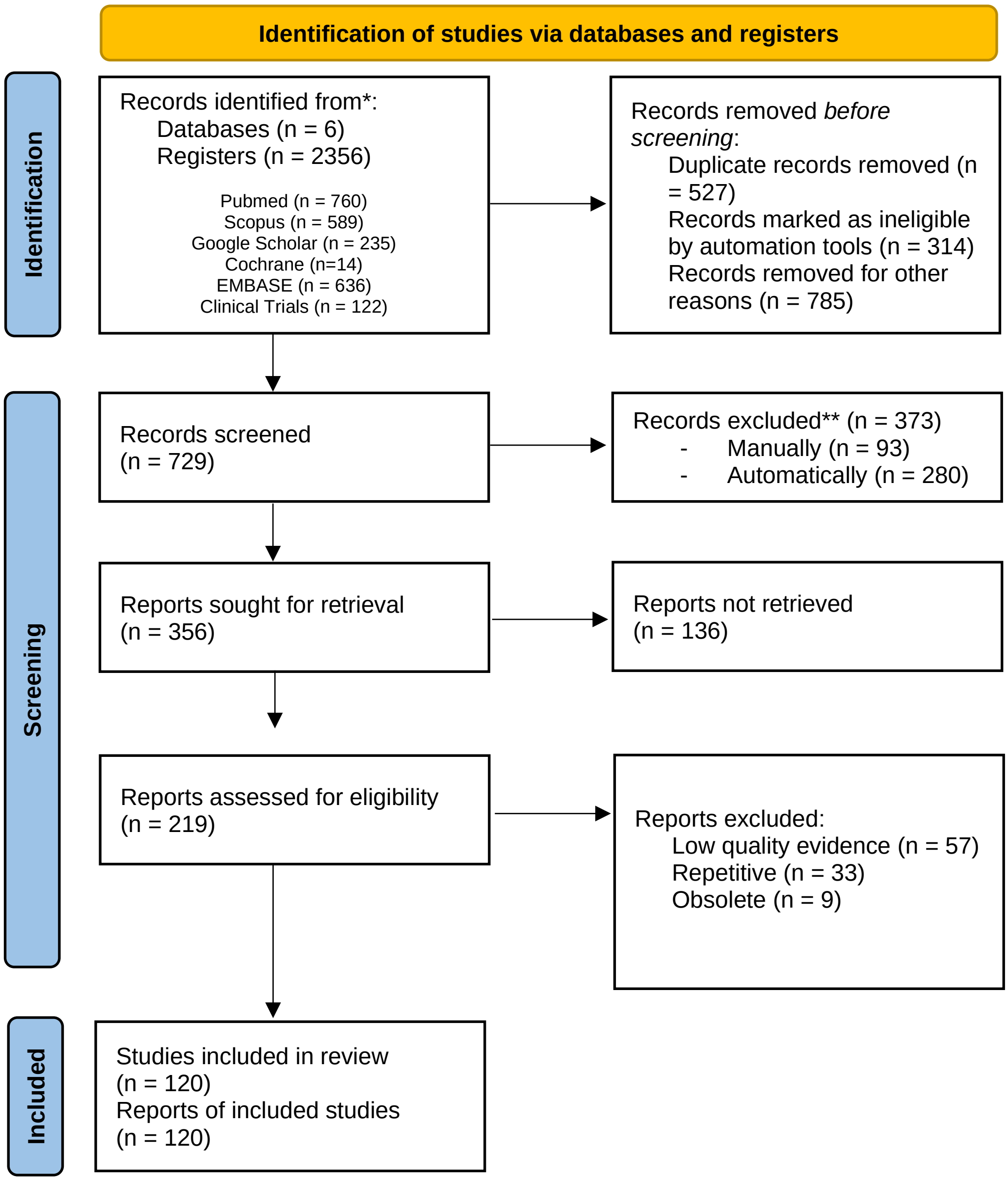

*Consider, if feasible to do so, reporting the number of records identified from each database or register searched (rather than the total number across all databases/registers).

**If automation tools were used, indicate how many records were excluded by a human and how many were excluded by automation tools.

From: Page MJ, McKenzie JE, Bossuyt PM, Boutron I, Hoffmann TC, Mulrow CD, et al. The PRISMA 2020 statement: an updated guideline for reporting systematic reviews. BMJ 2021;372:n71. doi: 10.1136/bmj.n71

For more information, visit: http://www.prisma-statement.org/
