# Supplementary material for: Rho-Kinase Inhibitors for the Treatment of Refractory Diabetic Macular Oedema
Source: Cells. 2021 Jul 3;10(7):1683. doi: 10.3390/cells10071683 (PMC8307715; doi:10.3390/cells10071683)
Supplement: Supplementary file 1 [file cells-10-01683-s001.zip › MMAT.pdf]

Supplementary File 2. Mixed Methods Appraisal Tool (MMAT)

RefID	First author	Year	Study design	Citation	SCREENING QUESTIONS		1. QUALITATIVE STUDIES					2. RANDOMIZED CONTROLLED TRIALS					3. NON-RANDOMIZED STUDIES					4. QUANTITATIVE DESCRIPTIVE STUDIES				
					S1. Are there clear research questions?	S2. Do the collected data allow to address the research questions?	1.1. Is the qualitative approach appropriate to answer the research question?	1.2. Are the qualitative data collection methods adequate to address the research question?	1.3. Are the findings adequately derived from the data?	1.4. Is the interpretation of results sufficiently substantiated by data?	1.5. Is there coherence between qualitative data sources, collection, analysis and interpretation?	2.1. Is randomization appropriately performed?	2.2. Are the groups comparable at baseline?	2.3. Are there complete outcome data?	2.4. Are outcome assessors blinded to the intervention provided?	2.5 Did the participants adhere to the assigned intervention?	3.1. Are the participants representative of the target population?	3.2. Are measurements appropriate regarding both the outcome and intervention (or exposure)?	3.3. Are there complete outcome data?	3.4. Are the confounders accounted for in the design and analysis?	3.5. During the study period, is the intervention administered (or exposure occurred) as intended?	4.1. Is the sampling strategy relevant to address the research question?	4.2. Is the sample representative of the target population?	4.3. Are the measurements appropriate?	4.4. Is the risk of nonresponse bias low?	4.5. Is the statistical analysis appropriate to answer the research question?
**DMO**																										
1	Ajlan	2016	Review	Ajlan, R.S.; Silva, P.S.; Sun, J.K. Vascular Endothelial Growth Factor and Diabetic Retinal Disease. *Seminars in Ophthalmology* **2016**, *31*, 40–48, doi:10.3109/08820538.2015.1114833.	Yes	Yes	Yes	CT	Yes	Yes	Yes															
2	Daruich	2018	Review	Daruich, A.; Matet, A.; Moulin, A.; Kowalczuk, L.; Nicolas, M.; Sellam, A.; Rothschild, P.R.; Omri, S.; Gélizé, E.; Jonet, L.; et al. *Mechanisms of Macular Edema: Beyond the Surface*; Elsevier Ltd, 2018; Vol. 63; ISBN 3314427816.	Yes	Yes	Yes	CT	Yes	Yes	Yes															
3	Rübsam	2018	Review	Rübsam, A.; Parikh, S.; Fort, P.E. Role of Inflammation in Diabetic Retinopathy. *International Journal of Molecular Sciences* 2018, *19*.	Yes	Yes	Yes	CT	Yes	Yes	Yes															
4	Nguyen	2012	RCT	Nguyen, Q.D.; Brown, D.M.; Marcus, D.M.; Boyer, D.S.; Patel, S.; Feiner, L.; Gibson, A.; Sy, J.; Rundle, A.C.; Hopkins, J.J.; et al. Ranibizumab for Diabetic Macular Edema: Results from 2 Phase Iii Randomized Trials: RISE and RIDE. *Ophthalmology* **2012**, *119*, 789–801, doi:10.1016/j.ophtha.2011.12.039.								Yes	Yes	Yes	Yes	Yes										
5	ETDRS	1991	RCT	Early Photocoagulation for Diabetic Retinopathy: ETDRS Report Number 9. *Ophthalmology* **1991**, *98*, 766–785, doi:10.1016/S0161-6420(13)38011-7.	Yes	Yes						Yes	Yes	Yes	Yes	Yes										
6	Heier	2016	RCT	Heier, J.S.; Korobelnik, J.F.; Brown, D.M.; Schmidt-Erfurth, U.; Do, D. v.; Midena, E.; Boyer, D.S.; Terasaki, H.; Kaiser, P.K.; Marcus, D.M.; et al. Intravitreal Aflibercept for Diabetic Macular Edema: 148-Week Results from the VISTA and VIVID Studies. In Proceedings of the Ophthalmology; Elsevier Inc., November 1 2016; Vol. 123, pp. 2376–2385.	Yes	Yes						Yes	Yes	Yes	Yes	Yes										
7	Arima	2020	Preclinical trial	Arima, M.; Nakao, S.; Yamaguchi, M.; Feng, H.; Fujii, Y.; Ishibashi, T.; Stitt, A.; Sonoda, K. Claudin-5 Redistribution Induced by Inflammation Leads to Anti- . Arima, M., Nakao, S., Yamaguchi, M., Feng, H., Fujii, Y., Ishibashi, T., … Sonoda, K. (2020). Diabetes, 69(5), 981–999. doi: 10.2337/db19-1121	Yes	Yes											Yes	Yes	Yes	Yes	Yes					
8	Nguyen	2019	RCT	Nguyen, Q.D.; Sepah, Y.J.; Berger, B.; Brown, D.; Do, D. v.; Garcia-Hernandez, A.; Patel, S.; Rahhal, F.M.; Shildkrot, Y.; Renfurm, R.W. Primary Outcomes of the VIDI Study: Phase 2, Double-Masked, Randomized, Active-Controlled Study of ASP8232 for Diabetic Macular Edema. *International Journal of Retina and Vitreous* **2019**, *5*, 1–14, doi:10.1186/s40942-019-0178-7.	Yes	Yes						Yes	Yes	Yes	Yes	Yes										

9	Miller	2018	Review	Miller, K.; Fortun, J.A. Diabetic Macular Edema: Current Understanding, Pharmacologic Treatment Options, and Developing Therapies. *Asia-Pacific Journal of Ophthalmology* **2018**, *7*, 28–35, doi:10.22608/APO.2017529.	Yes	Yes	Yes	CT	Yes	Yes	Yes					
10	Romero-Aroca	2016	Review	Romero-Aroca, P.; Baget-Bernaldiz, M.; Pareja-Rios, A.; Lopez-Galvez, M.; Navarro-Gil, R.; Verges, R. Diabetic Macular Edema Pathophysiology: Vasogenic versus Inflammatory. *Journal of Diabetes Research* **2016**, 2016:2156273 . doi:10.1155/2016/2156273	Yes	Yes	Yes	CT	Yes	Yes	Yes					
11	Tonade	2020	Review	Tonade, D.; Kern, T.S. Photoreceptor Cells and RPE Contribute to the Development of Diabetic Retinopathy. *Progress in Retinal and Eye Research* 2020, 100919. doi:10.1016/j.preteyeres.2020.100919	Yes	Yes	Yes	CT	Yes	Yes	Yes					
12	Bahrami	2016	Review	Bahrami, B.; Zhu, M.; Hong, T.; Chang, A. Diabetic Macular Oedema: Pathophysiology, Management Challenges and Treatment Resistance. *Diabetologia* 2016, *59*, 1594–1608. doi:10.1007/s00125-016-3974-8	Yes	Yes	Yes	CT	Yes	Yes	Yes					
13	Stitt	2005	Review	Stitt, A.; Frizzell, N.; Thorpe, S. Advanced Glycation and Advanced Lipoxidation: Possible Role in Initiation and Progression of Diabetic Retinopathy. *Current Pharmaceutical Design* **2005**, *10*, 3349–3360, doi:10.2174/1381612043383124.	Yes	Yes	Yes	CT	Yes	Yes	Yes					
14	Izumi	2003	Preclinical trial	Izumi, Y.; Hammerman, S.B.; Kirby, C.O.; Benz, A.M.; Olney, J.W.; Zorumski, C.F. Involvement of Glutamate in Ischemic Neurodegeneration in Isolated Retina. *Visual Neuroscience* **2003**, *20*, 97–107, doi:10.1017/S0952523803202017.	Yes	Yes						Yes	Yes	Yes	Yes	Yes
15	Fiorentino	2013	Review	Fiorentino, T.; Prioletta, A.; Zuo, P.; Folli, F. Hyperglycemia-Induced Oxidative Stress and Its Role in Diabetes Mellitus Related Cardiovascular Diseases. *Current Pharmaceutical Design* **2013**, *19*, 5695–5703, doi:10.2174/1381612811319320005.	Yes	Yes	Yes	CT	Yes	Yes	Yes					
16	Fong	2004	Review	Fong, D.S.; Aiello, L.; Gardner, T.W.; King, G.L.; Blankenship, G.; Cavallerano, J.D.; Ferris, F.L.; Klein, R. Retinopathy in Diabetes. *Diabetes Care* 2004, *27*.	Yes	Yes	Yes	CT	Yes	Yes	Yes					
17	Hussain	2016	Review	Hussain, R.M.; Ciulla, T.A. Treatment Strategies for Refractory Diabetic Macular Edema: Switching Anti-VEGF Treatments, Adopting Corticosteroid-Based Treatments, and Combination Therapy. *Expert Opinion on Biological Therapy* 2016, *16*, 365–374.	Yes	Yes	Yes	CT	Yes	Yes	Yes					
18	Das-Evcimen	2007	Review	das Evcimen, N.; King, G.L. The Role of Protein Kinase C Activation and the Vascular Complications of Diabetes. *Pharmacological Research* 2007, *55*, 498–510.	Yes	Yes	Yes	CT	Yes	Yes	Yes					

19	Araujo	2018	Review	Araújo, R.S.; Santos, D.F.; Silva, G.A. The Role of the Retinal Pigment Epithelium and Müller Cells Secretome in Neovascular Retinal Pathologies. *Biochimie* **2018**, *155*, 104–108, doi:10.1016/j.biochi.2018.06.019.	Yes	Yes	Yes	CT	Yes	Yes	Yes					
20	Rizzolo	2007	Review	Rizzolo, L.J. Development and Role of Tight Junctions in the Retinal Pigment Epithelium. *International Review of Cytology* **2007**, *258*, 195–234, doi:10.1016/S0074-7696(07)58004-6.	Yes	YEs	Yes	CT	Yes	Yes	Yes					
21	Omri	2010	Preclinical trial	Omri, S.; Omri, B.; Savoldelli, M.; Jonet, L.; Thillaye-Goldenberg, B.; Thuret, G.; Gain, P.; Jeanny, J.C.; Crisanti, P.; Behar-Cohen, F. The Outer Limiting Membrane (OLM) Revisited: Clinical Implications. *Clinical Ophthalmology* **2010**, *4*, 183–195, doi:10.2147/opth.s5901.	Yes	Yes						Yes	Yes	Yes	Yes	Yes
22	Sacconi	2019	Review	Sacconi, R.; Giuffrè, C.; Corbelli, E.; Borrelli, E.; Querques, G.; Bandello, F. Emerging Therapies in the Management of Macular Edema: A Review. F1000Res. 2019 Aug 12;8:F1000 Faculty Rev-1413. doi: 10.12688/f1000research.19198.1.	Yes	Yes	Yes	CT	Yes	Yes	Yes					
23	Miller	2013	Review	Miller, J.W.; le Couter, J.; Strauss, E.C.; Ferrara, N. Vascular Endothelial Growth Factor a in Intraocular Vascular Disease. *Ophthalmology* **2013**, *120*, 106–114, doi:10.1016/j.ophtha.2012.07.038.	Yes	Yes	Yes	CT	Yes	Yes	Yes					
24	Sodhi	2019	Preclinical trial	Sodhi, A.; Ma, T.; Menon, D.; Deshpande, M.; Jee, K.; Dinabandhu, A.; Vancel, J.; Lu, D.; Montaner, S. Angiopoietin-like 4 Binds Neuropilins and Cooperates with VEGF to Induce Diabetic Macular Edema. *Journal of Clinical Investigation* **2019**, *129*, 4593–4608, doi:10.1172/JCI120879.	Yes	Yes						Yes	Yes	Yes	Yes	Yes
25	Fogli	2016	Review	Fogli, S.; Mogavero, S.; Egan, C.G.; del Re, M.; Danesi, R. Pathophysiology and Pharmacological Targets of VEGF in Diabetic Macular Edema. *Pharmacological Research* **2016**, *103*, 149–157, doi:10.1016/j.phrs.2015.11.003.	Yes	Yes	Yes	CT	Yes	Yes	Yes					
26	Rizzolo	2011	Review	Rizzolo, L.J.; Peng, S.; Luo, Y.; Xiao, W. Integration of Tight Junctions and Claudins with the Barrier Functions of the Retinal Pigment Epithelium. *Progress in Retinal and Eye Research* 2011, *30*, 296–323.	Yes	Yes	Yes	CT	Yes	Yes	Yes					
27	Beranova-Giorgianni	2018	Review	Beranova-Giorgianni, S.; Giorgianni, F. Proteomics of Human Retinal Pigment Epithelium (RPE) Cells. *Proteomes* **2018**, *6*, 22, doi:10.3390/proteomes6020022.	Yes	Yes	Yes	CT	Yes	Yes	Yes					
28	Ponnalagu	2017	Review	Ponnalagu, M.; Subramani, M.; Jayadev, C.; Shetty, R.; Das, D. Retinal Pigment Epithelium-Secretome: A Diabetic Retinopathy Perspective. *Cytokine* 2017, *95*, 126–135.	Yes	Yes	Yes	CT	Yes	Yes	Yes					

29	Simo	2009	Review	Simó, R.; Villarroel, M.; Corraliza, L.; Hernández, C.; Garcia-Ramírez, M. The Retinal Pigment Epithelium: Something More than a Constituent of the Blood-Retinal Barrier-Implications for the Pathogenesis of Diabetic Retinopathy. *Journal of Biomedicine and Biotechnology* 2010, *2010*.	Yes	Yes	Yes	CT	Yes	Yes	Yes										
30	Rizzolo	2014	Review	Rizzolo, L.J. Barrier Properties of Cultured Retinal Pigment Epithelium. *Experimental Eye Research* 2014, *126*, 16–26.	Yes	Yes	Yes	CT	Yes	Yes	Yes										
31	Bonilha	2014	Review	Bonilha, V.L. Retinal Pigment Epithelium (RPE) Cytoskeleton Invivo and Invitro. *Experimental Eye Research* 2014, *126*, 38–45.	Yes	Yes	Yes	CT	Yes	Yes	Yes										
32	Jo	2019	Preclinical trial	Jo, D.H.; Yun, J.H.; Cho, C.S.; Kim, J.H.; Kim, J.H.; Cho, C.H. Interaction between Microglia and Retinal Pigment Epithelial Cells Determines the Integrity of Outer Blood-Retinal Barrier in Diabetic Retinopathy. *GLIA* **2019**, *67*, 321–331, doi:10.1002/glia.23542.	Yes	Yes						Yes	Yes	Yes	Yes	Yes					
33	Xu	2011	Review	Xu, H.Z.; Song, Z.; Fu, S.; Zhu, M.; Le, Y.Z. RPE Barrier Breakdown in Diabetic Retinopathy: Seeing Is Believing. *Journal of Ocular Biology, Diseases, and Informatics* **2011**, *4*, 83–92, doi:10.1007/s12177-011-9068-4.	Yes	Yes	Yes	CT	Yes	Yes	Yes										
34	Fu	2018	Preclinical trial	Fu, Z.; Wang, Z.; Liu, C.-H.; Gong, Y.; Cakir, B.; Liegl, R.; Sun, Y.; Meng, S.S.; Burnim, S.B.; Arellano, I.; et al. Fibroblast Growth Factor 21 Protects Photoreceptor Function in Type 1 Diabetic Mice. **2018**, doi:10.2337/db17-0830.	Yes	Yes						Yes	Yes	Yes	Yes	Yes					
35	Lammer	2016	Descriptive comparative study	Lammer, J.; Prager, S.G.; Cheney, M.C.; Ahmed, A.; Radwan, S.H.; Burns, S.A.; Silva, P.S.; Sun, J.K. Cone Photoreceptor Irregularity on Adaptive Optics Scanning Laser Ophthalmoscopy Correlates with Severity of Diabetic Retinopathy and Macular Edema. *Investigative Ophthalmology and Visual Science* **2016**, *57*, 6624–6632, doi:10.1167/iovs.16-19537.	Yes	Yes											Yes	Yes	Yes	Yes	Yes
36	Xia	2017	Review	Xia, T.; Rizzolo, L.J. Effects of Diabetic Retinopathy on the Barrier Functions of the Retinal Pigment Epithelium. *Vision Research* **2017**, *139*, 72–81, doi:10.1016/j.visres.2017.02.006.	Yes	Yes	Yes	CT	Yes	Yes	Yes										
37	Xu	2011	Preclinical trial	Xu, H.Z.; Le, Y.Z. Significance of Outer Blood-Retina Barrier Breakdown in Diabetes and Ischemia. *Investigative Ophthalmology and Visual Science* **2011**, *52*, 2160–2164, doi:10.1167/iovs.10-6518.	Yes	Yes						Yes	Yes	Yes	Yes	Yes					
38	Omri	2011	Preclinical trial	Omri, S.; Behar-Cohen, F.; de Kozak, Y.; Sennlaub, F.; Mafra Verissimo, L.; Jonet, L.; Savoldelli, M.; Omri, B.; Crisanti, P. Microglia/Macrophages Migrate through Retinal Epithelium Barrier by a Transcellular Route in Diabetic Retinopathy: Role of PKCζ in the Goto Kakizaki Rat Model. *American Journal of Pathology* **2011**, *179*, 942–953, doi:10.1016/j.ajpath.2011.04.018.	Yes	Yes						Yes	Yes	Yes	Yes	Yes					

39	Cunha-Vaz	2014	Review	Cunha-Vaz, J.; Ribeiro, L.; Lobo, C. Phenotypes and Biomarkers of Diabetic Retinopathy. *Progress in Retinal and Eye Research* 2014, *41*, 90–111. doi: 10.1016/j.preteyeres.2014.03.003	Yes	Yes	Yes	CT	Yes	Yes	Yes					
40	Al-Shabrawey	2008	Preclinical trial	Al-Shabrawey, M.; Rojas, M.; Sanders, T.; Behzadian, A.; El-Remessy, A.; Bartoli, M.; Parpia, A.K.; Liou, G.; Caldwell, R.B. Role of NADPH Oxidase in Retinal Vascular Inflammation. *Investigative Ophthalmology and Visual Science* **2008**, *49*, 3239–3244, doi:10.1167/iovs.08-1755.	Yes	Yes						Yes	Yes	Yes	Yes	Yes
41	Li	2012	Preclinical trial	Li, X.; Cai, Y.; Wang, Y.-S.; Shi, Y.-Y.; Hou, W.; Xu, C.-S.; Wang, H.-Y.; Ye, Z.; Yao, L.-B.; Zhang, J. Hyperglycaemia Exacerbates Choroidal Neovascularisation in Mice via the Oxidative Stress-Induced Activation of STAT3 Signalling in RPE Cells. *PLoS ONE* **2012**, *7*, e47600, doi:10.1371/journal.pone.0047600.	Yes	Yes						Yes	Yes	Yes	Yes	Yes
42	Huang	2011	Preclinical trial	Huang, H.; Gandhi, J.K.; Zhong, X.; Wei, Y.; Gong, J.; Duh, E.J.; Vinores, S.A. TNFα Is Required for Late BRB Breakdown in Diabetic Retinopathy, and Its Inhibition Prevents Leukostasis and Protects Vessels and Neurons from Apoptosis. *Investigative Ophthalmology and Visual Science* **2011**, *52*, 1336–1344, doi:10.1167/iovs.10-5768.	Yes	Yes						Yes	Yes	Yes	Yes	Yes
43	Zur	2019	Review	Zur, D.; Iglicki, M.; Loewenstein, A. The Role of Steroids in the Management of Diabetic Macular Edema. *Ophthalmic Research* 2019, *62*, 231–236, doi: 10.1159/000499540	Yes	Yes	Yes	CT	Yes	Yes	Yes					
44	Chawan-Saad	2019	Review	Chawan-Saad, J.; Wu, M.; Wu, A.; Wu, L. Corticosteroids for Diabetic Macular Edema. *Taiwan Journal of Ophthalmology* 2019, *9*, 233–242.	Yes	Yes	Yes	CT	Yes	Yes	Yes					
45	Monickaraj	2016	Preclinical trial	Monickaraj, F.; McGuire, P.G.; Franco Nitta, C.; Ghosh, K.; Das, A. Cathepsin D: An Mφ-Derived Factor Mediating Increased Endothelial Cell Permeability with Implications for Alteration of the Blood-Retinal Barrier in Diabetic Retinopathy. *FASEB Journal* **2016**, *30*, 1670–1682, doi:10.1096/fj.15-279802.	Yes	Yes						Yes	Yes	Yes	Yes	Yes
46	Hussain	2019	Review	Hussain, R.M.; Neiweem, A.E.; Kansara, V.; Harris, A.; Ciulla, T.A. Tie-2/Angiopoietin Pathway Modulation as a Therapeutic Strategy for Retinal Disease. *Expert Opinion on Investigational Drugs* 2019, *28*, 861–869.	Yes	Yes	Yes	CT	YEs	Yes	Yes					
47	Joussen	2001	Preclinical trial	Joussen, A.M.; Murata, T.; Tsujikawa, A.; Kirchhof, B.; Bursell, S.E.; Adamis, A.P. Leukocyte-Mediated Endothelial Cell Injury and Death in the Diabetic Retina. *American Journal of Pathology* **2001**, *158*, 147–152, doi:10.1016/S0002-9440(10)63952-1.	Yes	Yes						Yes	Yes	Yes	Yes	Yes
48	Ciulla	2003	Review	Ciulla, T.A.; Amador, A.G.; Zinman, B. *Diabetic Retinopathy and Diabetic Macular Edema Pathophysiology, Screening, and Novel Therapies*; 2003;	Yes	Yes	Yes	CT	Yes	Yes	Yes					

49	Joussen	2004	Preclinical trial	Joussen, A.M.; Poulaki, V.; Le, M.L.; Koizumi, K.; Esser, C.; Janicki, H.; Schraermeyer, U.; Kociok, N.; Fauser, S.; Kirchhof, B.; et al. A Central Role for Inflammation in the Pathogenesis of Diabetic Retinopathy. *The FASEB Journal* **2004**, *18*, 1450–1452, doi:10.1096/fj.03-1476fje.	Yes	Yes											Yes	Yes	Yes	Yes	Yes					
50	Bressler	2018	RCT	Bressler, N.M.; Beaulieu, W.T.; Glassman, A.R.; Blinder, K.J.; Bressler, S.B.; Jampol, L.M.; Melia, M.; Wells, J.A. Persistent Macular Thickening Following Intravitreous Aflibercept, Bevacizumab, or Ranibizumab for Central-Involved Diabetic Macular Edema with Vision Impairment A Secondary Analysis of a Randomized Clinical Trial. In Proceedings of the JAMA Ophthalmology; American Medical Association, March 1 2018; Vol. 136, pp. 257–269.	Yes	Yes						Yes	Yes	Yes	Yes	Yes										
51	Iglicki	2019	Comparative retrospective study	Iglicki, M.; Busch, C.; Zur, D.; Okada, M.; Mariussi, M.; Chhablani, J.K.; Cebeci, Z.; Fraser-Bell, S.; Chaikitmongkol, V.; Couturier, A.; et al. Dexamethasone Implant for Diabetic Macular Edema in Naive Compared with Refractory Eyes: The International Retina Group Real-Life 24-Month Multicenter Study: The Irgrel-Dex Study. *Retina* **2019**, *39*, 44–51, doi:10.1097/IAE.0000000000002196.	Yes	Yes											Yes	Yes	Yes	Yes	Yes					
52	Maturi	2017	Review	Shah, S.U.; Maturi, R.K. Therapeutic Options in Refractory Diabetic Macular Oedema. *Drugs* **2017**, *77*, 481–492, doi:10.1007/s40265-017-0704-6.	Yes	Yes	Yes	CT	Yes	Yes	Yes															
53	Virgili	2018	Meta-analysis	Virgili, G.; Parravano, M.; Evans, J.R.; Gordon, I.; Lucenteforte, E. Anti-Vascular Endothelial Growth Factor for Diabetic Macular Oedema: A Network Meta-Analysis. *Cochrane Database of Systematic Reviews* 2018, *2018*.	Yes	Yes	Yes	Yes	Yes	Yes	Yes															
54	The DRCRN	2015	RCT	Aflibercept, Bevacizumab, or Ranibizumab for Diabetic Macular Edema. *New England Journal of Medicine* **2015**, *372*, 1193–1203, doi:10.1056/nejmoa1414264.	Yes	Yes						Yes	Yes	YEs	Yes	Yes										
55	Karst	2019	Descriptive retrospective study	Karst, S.G.; Schuster, M.; Mitsch, C.; Meyer, E.L.; Kundi, M.; Scholda, C.; Schmidt-Erfurth, U.M. Atrophy of the Central Neuroretina in Patients Treated for Diabetic Macular Edema. *Acta Ophthalmologica* **2019**, *97*, e1054–e1061, doi:10.1111/aos.14173.	Yes	Yes																Yes	Yes	Yes	Yes	Yes
56	Willmann	2017	Comparative retrospective study	Willmann, G.; Nepomuceno, A.B.; Messias, K.; Barroso, L.; Scott, I.U.; Messias, A.; Jorge, R. Foveal Thickness Reduction after Anti-Vascular Endothelial Growth Factor Treatment in Chronic Diabetic Macular Edema. *International Journal of Ophthalmology* **2017**, *10*, 760–764, doi:10.18240/ijo.2017.05.17.	Yes	Yes											Yes	Yes	Yes	Yes	Yes					

57	Romano	2019	Review	Romano, M.R.; Allegrini, D.; Della Guardia, C.; Schiemer, S.; Baronissi, I.; Ferrara, M.; Cennamo, G. Vitreous and Intraretinal Macular Changes in Diabetic Macular Edema with and without Tractional Components. *Graefe's Archive for Clinical and Experimental Ophthalmology* 2019, *257*, 1–8.	Yes	Yes	Yes	CT	Yes	Yes	Yes					
58	Ferris	2017	Comparative retrospective study	Ferris, F.L.; Maguire, M.G.; Glassman, A.R.; Ying, G.S.; Martin, D.F. Evaluating Effects of Switching Anti-Vascular Endothelial Growth Factor Drugs for Age-Related Macular Degeneration and Diabetic Macular Edema. *JAMA Ophthalmology* **2017**, *135*, 145–149, doi:10.1001/jamaophthalmol.2016.4820.	Yes	Yes						Yes	Yes	Yes	Yes	Yes
59	Dhoot	2015	Comparative Prospective study	Dhoot, D.S.; Pieramici, D.J.; Nasir, M.; Castellarin, A.A.; Couvillion, S.; See, R.F.; Steinle, N.; Bennett, M.; Rabena, M.; Avery, R.L. Residual Edema Evaluation with Ranibizumab 0.5 Mg and 2.0 Mg Formulations for Diabetic Macular Edema (REEF Study). *Eye (Basingstoke)* **2015**, *29*, 534–541, doi:10.1038/eye.2014.338.	Yes	Yes						Yes	Yes	Yes	Yes	Yes
60	Ciulla	2016	Review	Ciulla, T.A.; Hussain, R.M.; Ciulla, L.M.; Sink, B.; Harris, A. Ranibizumab for Diabetic Macular Edema Refractory to Multiple Prior Treatments. *Retina* **2016**, *36*, 1292–1297, doi:10.1097/IAE.0000000000000876.	Yes	Yes	Yes	Yes	Yes	Yes	Yes					
61	Wood	2015	Comparative prospective study	Wood, E.H.; Karth, P.A.; Moshfeghi, D.M.; Leng, T. Short-Term Outcomes of Aflibercept Therapy for Diabetic Macular Edema in Patients with Incomplete Response to Ranibizumab and/or Bevacizumab. In Proceedings of the Ophthalmic Surgery Lasers and Imaging Retina; Slack Incorporated, October 1 2015; Vol. 46, pp. 950–954.	Yes	Yes						Yes	Yes	Yes	Yes	Yes
62	Khan	2017	Meta-analysis	Khan, Z.; Kuriakose, R.K.; Khan, M.; Chin, E.K.; Almeida, D.R.P. Effcacy of the Intravitreal Sustained-Release Dexamethasone Implant for Diabetic Macular Edema Refractory to Anti-Vascular Endothelial Growth Factor Therapy: Meta-Analysis and Clinical Implications. *Ophthalmic Surgery Lasers and Imaging Retina* **2017**, *48*, 160–166, doi:10.3928/23258160-20170130-10.	Yes	Yes	Yes	Yes	Yes	Yes	Yes					
63	Estebainha	2020	Review	Estebainha, R.; Goldhardt, R.; Falcão, M. A New Approach for Diabetic Macular Edema Treatment: Review of Clinical Practice Results with 0.19 Mg Fluocinolone Acetonide Intravitreal Implant Including Vitrectomized Eyes. *Current Ophthalmology Reports* 2020, *8*, 1–10.	Yes	Yes	Yes	Yes	Yes	Yes	Yes					
64	He	2018	Meta-analysis	He, Y.; Ren, X.J.; Hu, B.J.; Lam, W.C.; Li, X.R. A Meta-Analysis of the Effect of a Dexamethasone Intravitreal Implant versus Intravitreal Anti-Vascular Endothelial Growth Factor Treatment for Diabetic Macular Edema. *BMC Ophthalmology* **2018**, *18*, 121, doi:10.1186/s12886-018-0779-1.	Yes	Yes	Yes	Yes	Yes	Yes	Yes					

65	Mehta	2018	Systematic Review	Mehta, H.; Hennings, C.; Gillies, M.C.; Nguyen, V.; Campain, A.; Fraser-Bell, S. Anti-Vascular Endothelial Growth Factor Combined with Intravitreal Steroids for Diabetic Macular Oedema. *Cochrane Database of Systematic Reviews* **2018**, *2018*, doi:10.1002/14651858.CD011599.pub2.	Yes	Yes	Yes	Yes	Yes	Yes	Yes										
66	Boyer	2014	RCT	Boyer, D.S.; Yoon, Y.H.; Belfort, R.; Bandello, F.; Maturi, R.K.; Augustin, A.J.; Li, X.Y.; Cui, H.; Hashad, Y.; Whitcup, S.M. Three-Year, Randomized, Sham-Controlled Trial of Dexamethasone Intravitreal Implant in Patients with Diabetic Macular Edema. *Ophthalmology* **2014**, *121*, 1904–1914, doi:10.1016/j.ophtha.2014.04.024.	Yes	Yes						Yes	Yes	Yes	Yes	Yes					
67	Maturi	2017	RCT	Maturi, R.K.; Glassman, A.R.; Liu, D.; Beck, R.W.; Bhavsar, A.R.; Bressler, N.M.; Jampol, L.M.; Melia, M.; Punjabi, O.S.; Salehi-Had, H.; et al. Effect of Adding Dexamethasone to Continued Ranibizumab Treatment in Patients with Persistent Diabetic Macular Edema: A DRCR Network Phase 2 Randomized Clinical Trial. *JAMA Ophthalmology* **2018**, *136*, 29–38, doi:10.1001/jamaophthalmol.2017.4914.	Yes	Yes						Yes	Yes	Yes	Yes	Yes					
68	Stefánsson	2001	Review	Stefánsson, E. The Therapeutic Effects of Retinal Laser Treatment and Vitrectomy. A Theory Based on Oxygen and Vascular Physiology. *Acta Ophthalmologica Scandinavica* 2001, *79*, 435–440.	Yes	Yes	Yes	CT	Yes	Yes	Yes										
69	Chin	2005	Preclinical trial	Chin, H.S.; Park, T.S.; Moon, Y.S.; Oh, J.H. Difference in Clearance of Intravitreal Triamcinolone Acetonide between Vitrectomized and Nonvitrectomized Eyes. *Retina* **2005**, *25*, 556–560, doi:10.1097/00006982-200507000-00002.	Yes	Yes											Yes	Yes	Yes	Yes	Yes
70	Yau	2012	Systematic Review	Yau, J.W.Y.; Rogers, S.L.; Kawasaki, R.; Lamoureux, E.L.; Kowalski, J.W.; Bek, T.; Chen, S.J.; Dekker, J.M.; Fletcher, A.; Grauslund, J.; et al. Global Prevalence and Major Risk Factors of Diabetic Retinopathy. *Diabetes Care* **2012**, *35*, 556–564, doi:10.2337/dc11-1909.	Yes	Yes	Yes	Yes	Yes	Yes	Yes										
**ROCK**																					
71	Ruiz-Loredo	2011	Preclinical trial	Ruiz-Loredo, A.Y.; López, E.; López-Colomé, A.M. Thrombin Promotes Actin Stress Fiber Formation in RPE through Rho/ROCK-Mediated MLC Phosphorylation. *Journal of Cellular Physiology* **2011**, *226*, 414–423, doi:10.1002/jcp.22347.	Yes	Yes											Yes	Yes	Yes	Yes	Yes
72	Jahani	2018	Review	Jahani, V.; Kavousi, A.; Mehri, S.; Karimi, G. Rho Kinase, a Potential Target in the Treatment of Metabolic Syndrome. *Biomedicine and Pharmacotherapy* 2018, *106*, 1024–1030.	Yes	Yes	Yes	CT	Yes	Yes	Yes										

73	Moshifar	2018	Review	Moshirfar, M.; Parker, L.; Birdsong, O.C.; Ronquillo, Y.C.; Hofstedt, D.; Shah, T.J.; Gomez, A.T.; Hoopes, P.C.S. Use of Rho Kinase Inhibitors in Ophthalmology: A Review of the Literature. *Medical hypothesis, discovery & innovation ophthalmology journal* **2018**, *7*, 101–111.	Yes	Yes	Yes	CT	Yes	Yes	Yes					
74	Rao	2017	Review	Rao, P.V.; Pattabiraman, P.P.; Kopczynski, C. Role of the Rho GTPase/Rho Kinase Signalling Pathway in Pathogenesis and Treatment of Glaucoma: Bench to Bedside Research. *Experimental Eye Research* 2017, *158*, 23–32.	Yes	Yes	Yes	CT	Yes	Yes	Yes					
75	Feng	2016	Review	Feng, Y.; Lograsso, P. v.; Defert, O.; Li, R. Rho Kinase (ROCK) Inhibitors and Their Therapeutic Potential. *Journal of Medicinal Chemistry* **2016**, *59*, 2269–2300, doi:10.1021/acs.jmedchem.5b00683.	Yes	Yes	Yes	CT	Yes	Yes	Yes					
76	Koch	2018	Review	Koch, J.C.; Tatenhorst, L.; Roser, A.E.; Saal, K.A.; Tönges, L.; Lingor, P. ROCK Inhibition in Models of Neurodegeneration and Its Potential for Clinical Translation. *Pharmacology and Therapeutics* **2018**, *189*, 1–21, doi:10.1016/j.pharmthera.2018.03.008.	Yes	Yes	Yes	CT	Yes	Yes	Yes					
77	Yamashita	2015	Preclinical trial	Yamashita, F.; Fukuyama, E.; Mizoguchi, K.; Takizawa, S.; Xu, S.; Kawakata, H. Scale Dependence of Rock Friction at High Work Rate. *Nature* **2015**, *528*, 254–257, doi:10.1038/nature16138.	Yes	Yes						Yes	Yes	Yes	Yes	Yes
78	Shimizu	2014	Comparative retrospective study	Shimizu, Y.; Dobashi, K.; Sano, T.; Yamada, M. Rock Activation in Lung of Idiopathic Pulmonary Fibrosis with Oxidative Stress. *International Journal of Immunopathology and Pharmacology* **2014**, *27*, 37–44, doi:10.1177/039463201402700106.	Yes	Yes						Yes	Yes	Yes	Yes	Yes
79	Zhang	2014	Preclinical trial	Zhang, C.; Zhang, S.; Zhang, Z.; He, J.; Xu, Y.; Liu, S. ROCK Has a Crucial Role in Regulating Prostate Tumor Growth through Interaction with C-Myc. *Oncogene* **2014**, *33*, 5582–5591, doi:10.1038/onc.2013.505.	Yes	YEs						Yes	Yes	Yes	Yes	Yes
80	Alleaume-butauz	2015	Preclinical trial	Alleaume-Butaux, A.; Nicot, S.; Pietri, M.; Baudry, A.; Dakowski, C.; Tixador, P.; Ardila-Osorio, H.; Haeberlé, A.M.; Bailly, Y.; Peyrin, J.M.; et al. Double-Edge Sword of Sustained ROCK Activation in Prion Diseases through Neuritogenesis Defects and Prion Accumulation. *PLoS Pathogens* **2015**, *11*, doi:10.1371/journal.ppat.1005073.	Yes	Yes						Yes	Yes	Yes	Yes	Yes
81	Halász	2016	Review	Halász, É.; Townes-Anderson, E. Rock Inhibitors in Ocular Disease. *ADMET and DMPK* 2016, *4*, 280–301.	Yes	Yes	Yes	CT	Yes	Yes	Yes					
82	Abbhi	2018	Review	Abbhi, V.; Piplani, P. Rho-Kinase (ROCK) Inhibitors - A Neuroprotective Therapeutic Paradigm with a Focus on Ocular Utility. *Current Medicinal Chemistry* **2018**, *27*, 2222–2256, doi:10.2174/0929867325666181031102829.	Yes	Yes	Yes	CT	Yes	Yes	Yes					

83	Fukiage	2001	Preclinical trial	Fukiage, C.; Mizutani, K.; Kawamoto, Y.; Azuma, M.; Shearer, T.R. Involvement of Phosphorylation of Myosin Phosphatase by ROCK in Trabecular Meshwork and Ciliary Muscle Contraction. *Biochemical and Biophysical Research Communications* **2001**, *288*, 296–300, doi:10.1006/bbrc.2001.5751.	Yes	Yes						Yes	Yes	Yes	Yes	Yes					
84	Sun	2006	Preclinical trial	Sun, H.; Breslin, J.W.; Zhu, J.; Yuan, S.Y.; Wu, M.H. Rho and ROCK Signalling in VEGF-Induced Microvascular Endothelial Hyperpermeability. *Microcirculation* **2006**, *13*, 237–247, doi:10.1080/10739680600556944.	Yes	Yes						Yes	Yes	Yes	Yes	Yes					
85	Nourinia	2018	Review	Nourinia, R.; Nakao, S.; Zandi, S.; Safi, S.; Hafezi-Moghadam, A.; Ahmadieh, H. ROCK Inhibitors for the Treatment of Ocular Diseases. *British Journal of Ophthalmology* 2018, *102*.	Yes	Yes	Yes	CT	Yes	Yes	Yes										
86	Arita	2010	Review	Arita, R.; Hata, Y.; Ishibashi, T. ROCK as a Therapeutic Target of Diabetic Retinopathy. *Journal of Ophthalmology* **2010**, *2010*, 1–9, doi:10.1155/2010/175163.	Yes	Yes	Yes	CT	Yes	Yes	Yes										
87	Lu	2014	Preclinical trial	Lu, Q.Y.; Chen, W.; Lu, L.; Zheng, Z.; Xu, X. Involvement of RhoA/ROCK1 Signalling Pathway in Hyperglycemia-Induced Microvascular Endothelial Dysfunction in Diabetic Retinopathy. *International Journal of Clinical and Experimental Pathology* **2014**, *7*, 7268–7277.	Yes	Yes						Yes	Yes	Yes	Yes	Yes					
88	Mohammad	2018	Preclinical trial	Mohammad, G.; AlSharif, H.M.; Siddiquei, M.M.; Ahmad, A.; Alam, K.; El-Asrar, A.M.A. Rho-Associated Protein Kinase-1 Mediates the Regulation of Inflammatory Markers in Diabetic Retina and in Retinal Müller Cells. *Annals of Clinical and Laboratory Science* **2018**, *48*, 137–145.	Yes	Yes						Yes	Yes	Yes	Yes	Yes					
89	Yao	2018	Preclinical trial	Yao, J.; Wang, J.; Yao, Y.; Wang, K.; Zhou, Q.; Tang, Y. MiR-133b Regulates Proliferation and Apoptosis in High-glucose-induced Human Retinal Endothelial Cells by Targeting Ras Homolog Family Member A. *International Journal of Molecular Medicine* **2018**, *42*, 839–850, doi:10.3892/ijmm.2018.3694.	Yes	Yes						Yes	Yes	Yes	Yes	Yes					
90	Ozturk	2009	Descriptive comparative study	Ozturk, B.T.; Bozkurt, B.; Kerimoglu, H.; Okka, M.; Kamis, U.; Gunduz, K. Effect of Serum Cytokines and VEGF Levels on Diabetic Retinopathy and Macular Thickness. *Molecular Vision* **2009**, *15*, 1906–1914.	Yes	Yes											Yes	Yes	Yes	Yes	Yes
91	Yamaguchi	2016	Preclinical trial	Yamaguchi, M.; Nakao, S.; Arita, R.; Kaizu, Y.; Arima, M.; Zhou, Y.; Kita, T.; Yoshida, S.; Kimura, K.; Isobe, T.; et al. Vascular Normalization by ROCK Inhibitor: Therapeutic Potential of Ripasudil (K-115) Eye Drop in Retinal Angiogenesis and Hypoxia. *Investigative Ophthalmology and Visual Science* **2016**, *57*, 2264–2276, doi:10.1167/iovs.15-17411.	Yes	Yes						Yes	Yes	Yes	Yes	Yes					

92	Bryan	2010	Preclinical trial	Bryan, B.A.; Dennstedt, E.; Mitchell, D.C.; Walshe, T.E.; Noma, K.; Loureiro, R.; Saint-Geniez, M.; Campaigniac, J.; Liao, J.K.; Patricia, D.A. RhoA/ROCK Signalling Is Essential for Multiple Aspects of VEGF-mediated Angiogenesis. *The FASEB Journal* **2010**, *24*, 3186–3195, doi:10.1096/fj.09-145102.	Yes	Yes	Yes	Yes	Yes	Yes	Yes
93	Zhang	2018	Preclinical trial	Zhang, X.H.; Feng, Z.H.; Wang, X.Y. The ROCK Pathway Inhibitor Y-27632 Mitigates Hypoxia and Oxidative Stress-Induced Injury to Retinal Muller Cells. *Neural Regeneration Research* **2018**, *13*, 549–555, doi:10.4103/1673-5374.228761.	Yes	Yes	Yes	Yes	Yes	Yes	Yes
94	Yokota	2007	Preclinical trial	Yokota, T.; Utsunomiya, K.; Taniguchi, K.; Gojo, A.; Kurata, H.; Tajima, N. Involvement of the Rho/Rho Kinase Signalling Pathway in Platelet-Derived Growth Factor BB-Induced Vascular Endothelial Growth Factor Expression in Diabetic Rat Retina. *Japanese Journal of Ophthalmology* **2007**, *51*, 424–430, doi:10.1007/s10384-007-0471-0.	Yes	Yes	Yes	Yes	Yes	Yes	Yes
95	Rothschild	2017	Preclinical trial	Rothschild, P.R.; Salah, S.; Berdugo, M.; Gélizé, E.; Delaunay, K.; Naud, M.C.; Klein, C.; Moulin, A.; Savoldelli, M.; Bergin, C.; et al. ROCK-1 Mediates Diabetes-Induced Retinal Pigment Epithelial and Endothelial Cell Blebbing: Contribution to Diabetic Retinopathy. *Scientific Reports* **2017**, *7*, 1–15, doi:10.1038/s41598-017-07329-y.	Yes	Yes	Yes	Yes	Yes	Yes	Yes
96	Arita	2009	Preclinical trial	Arita, R.; Hata, Y.; Nakao, S.; Kita, T.; Miura, M.; Kawahara, S.; Zandi, S.; Almulki, L.; Tayyari, F.; Shimokawa, H.; et al. Rho Kinase Inhibition by Fasudil Ameliorates Diabetes-Induced Microvascular Damage. *Diabetes* **2009**, *58*, 215–226, doi:10.2337/db08-0762.	Yes	Yes	Yes	Yes	Yes	Yes	Yes
97	Chen	2018	Preclinical trial	Chen, Y.L.; Ren, Y.; Xu, W.; Rosa, R.H.; Kuo, L.; Hein, T.W. Constriction of Retinal Venules to Endothelin-1: Obligatory Roles of ET A Receptors, Extracellular Calcium Entry, and Rho Kinase. *Investigative Ophthalmology and Visual Science* **2018**, *59*, 5167–5175, doi:10.1167/iovs.18-25369.	Yes	Yes	Yes	Yes	Yes	Yes	Yes
98	Rosa	2009	Preclinical trial	Rosa, R.H. Divergent Roles of Nitric Oxide and Rho Kinase in Vasomotor Regulation of Human Retinal Arterioles. **2009**, doi:10.1167/iovs.09-4391.	Yes	Yes	Yes	Yes	Yes	Yes	Yes
99	Yamaguchi	2016	Preclinical trial	Yamaguchi, M.; Nakao, S.; Arita, R.; Kaizu, Y.; Arima, M.; Zhou, Y.; Kita, T.; Yoshida, S.; Kimura, K.; Isobe, T.; et al. Vascular Normalization by ROCK Inhibitor: Therapeutic Potential of Ripasudil (K-115) Eye Drop in Retinal Angiogenesis and Hypoxia. *Investigative Ophthalmology and Visual Science* **2016**, *57*, 2264–2276, doi:10.1167/iovs.15-17411.	Yes	Yes	Yes	Yes	Yes	Yes	Yes
100	Nagaoka	2007	Preclinical trial	Nagaoka, T.; Hein, T.W.; Yoshida, A.; Kuo, L. Simvastatin Elicits Dilation of Isolated Porcine Retinal Arterioles: Role of Nitric Oxide and Mevalonate-Rho Kinase Pathways., doi:10.1167/iovs.06-0856.	Yes	Yes	Yes	Yes	Yes	Yes	Yes

101	Fagerholm	2019	Review	Fagerholm, S.C.; Guenther, C.; Asens, M.L.; Savinko, T.; Uotila, L.M. Beta2-Integins and Interacting Proteins in Leukocyte Trafficking, Immune Supression, and Immunodeficiency Disease. *Frontiers in Immunology* **2019**, *10*, 1–10, doi:10.3389/fimmu.2019.00254.	Yes	Yes	Yes	CT	Yes	Yes	Yes										
102	Barreiro	2002	Preclinical trial	Barreiro, O.; Yáñez-Mó, M.; Serrador, J.M.; Montoya, M.C.; Vicente-Manzanares, M.; Tejedor, R.; Furthmayr, H.; Sánchez-Madrid, F. Dynamic Interaction of VCAM-1 and ICAM-1 with Moesin and Ezrin in a Novel Endothelial Docking Structure for Adherent Leukocytes. *Journal of Cell Biology* **2002**, *157*, 1233–1245, doi:10.1083/jcb.200112126.	Yes	Yes											Yes	Yes	Yes	Yes	Yes
103	Nourinia	2013	RCT	Nourinia, R.; Ahmadieh, H.; Shahheidari, M.H.; Zandi, S.; Nakao, S.; Hafezi-Moghadam, A. Intravitreal Fasudil Combined with Bevacizumab for Treatment of Refractory Diabetic Macular Edema; a Pilot Study. *Journal of Ophthalmic and Vision Research* **2013**, *8*, 337–340.	Yes	Yes						Yes	Yes	Yes	Yes	Yes					
104	Ahmadieh	2013	Prospective interventional case serie	Ahmadieh, H.; Nourinia, R.; Hafezi-Moghadam, A. Intravitreal Fasudil Combined with Bevacizumab for Persistent Diabetic Macular Edema: A Novel Treatment. *JAMA Ophthalmology* **2013**, *131*, 923–924, doi:10.1001/jamaophthalmol.2013.143.	Yes	Yes											Yes	Yes	Yes	Yes	Yes
105	Van-Linden	2014	Review	van Linden, O.P.J.; Kooistra, A.J.; Leurs, R.; de Esch, I.J.P.; de Graaf, C. KLIFS: A Knowledge-Based Structural Database to Navigate Kinase-Ligand Interaction Space. *Journal of Medicinal Chemistry* 2014, *57*, 249–277.	Yes	Yes	Yes	Yes	Yes	Yes	Yes										
106	Zhao	2014	Review	Zhao, Z.; Wu, H.; Wang, L.; Liu, Y.; Knapp, S.; Liu, Q.; Gray, N.S. Exploration of Type II Binding Mode: A Privileged Approach for Kinase Inhibitor Focused Drug Discovery? *ACS Chemical Biology* 2014, *9*, 1230–1241.	Yes	Yes	Yes	CT	Yes	Yes	Yes										
107	Tanna	2018	Review	Tanna, A.P.; Johnson, M. Rho Kinase Inhibitors as a Novel Treatment for Glaucoma and Ocular Hypertension. *Ophthalmology* 2018, *125*, 1741–1756.	Yes	Yes	Yes	CT	Yes	Yes	Yes										
108	Hoy	2018	Review	Hoy, S.M. Netarsudil Ophthalmic Solution 0.02%: First Global Approval. *Drugs* **2018**, *78*, 389–396, doi:10.1007/s40265-018-0877-7.	Yes	Yes	Yes	CT	Yes	Yes	Yes										
109	Jagasia	2021	CT (Phase IIa)	Jagasia, M.; Lazaryan, A.; Bachier, C.R.; Salhotra, A.; Weisdorf, D.J.; Zoghi, B.; Essell, J.; Green, L.; Schueller, O.; Patel, J.; et al. ROCK2 Inhibition With Belumosudil (KD025) for the Treatment of Chronic Graft-Versus-Host Disease. *Journal of Clinical Oncology* **2021**, JCO.20.02754, doi:10.1200/jco.20.02754.	Yes	Yes											Yes	Yes	Yes	Yes	Yes
110	Garnock-Jones	2014	Review	Garnock-Jones, K.P. Ripasudil: First Global Approval. *Drugs* **2014**, *74*, 2211–2215, doi:10.1007/s40265-014-0333-2.	Yes	Yes	Yes	CT	Yes	Yes	Yes										

111	Nakabayashi	2015	Preclinical trial	Nakabayashi, S.; Kawai, M.; Yoshioka, T.; Song, Y.S.; Tani, T.; Yoshida, A.; Nagaoka, T. Effect of Intravitreal Rho Kinase Inhibitor Ripasudil (K-115) on Feline Retinal Microcirculation. *Experimental Eye Research* **2015**, *139*, 132–135, doi:10.1016/j.exer.2015.07.008.	Yes	Yes											Yes	Yes	Yes	Yes	Yes
112	Pakravan	2016	Prospective Interventional case serie	Pakravan, M.; Beni, A.N.; Ghahari, E.; Varshochian, R.; Yazdani, S.; Esfandiari, H.; Ahmadieh, H. The Ocular Hypotensive Efficacy of Topical Fasudil, a Rho-Associated Protein Kinase Inhibitor, in Patients with End-Stage Glaucoma. *American Journal of Therapeutics* **2016**, *24*, E676–E680, doi:10.1097/MJT.0000000000000362.	Yes	Yes											Yes	Yes	Yes	Yes	Yes
113	Mietzner	2019	Review	Mietzner, R.; Breunig, M. Causative Glaucoma Treatment: Promising Targets and Delivery Systems. *Drug Discovery Today* 2019, *24*, 1606–1613.	Yes	Yes	Yes	CT	Yes	Yes	Yes										
114	Li	2013	Preclinical trial	Li, M.; Yasumura, D.; Ma, A.A.K.; Matthes, M.T.; Yang, H.; Nielson, G.; Huang, Y.; Szoka, F.C.; LaVail, M.M.; Diamond, M.I. Intravitreal Administration of HA-1077, a ROCK Inhibitor, Improves Retinal Function in a Mouse Model of Huntington Disease. *PLoS ONE* **2013**, *8*, doi:10.1371/journal.pone.0056026.	Yes	Yes											Yes	Yes	Yes	Yes	Yes
115	Mietzner	2020	Preclinical trial	Mietzner, R.; Kade, C.; Froemel, F.; Pauly, D.; Stamer, W.D.; Ohlmann, A.; Wegener, J.; Fuchshofer, R.; Breunig, M. Fasudil Loaded PLGA Microspheres as Potential Intravitreal Depot Formulation for Glaucoma Therapy. *Pharmaceutics* **2020**, *12*, 1–22, doi:10.3390/pharmaceutics12080706.	Yes	Yes											Yes	Yes	Yes	Yes	Yes
116	Kita	2008	Preclinical trial	Kita, T.; Hata, Y.; Arita, R.; Kawahara, S.; Miura, M.; Nakao, S.; Mochizuki, Y.; Enaida, H.; Goto, Y.; Shimokawa, H.; et al. Role of TGF-β in Proliferative Vitreoretinal Diseases and ROCK as a Therapeutic Target. *Proceedings of the National Academy of Sciences of the United States of America* **2008**, *105*, 17504–17509, doi:10.1073/pnas.0804054105.	Yes	Yes											Yes	Yes	Yes	Yes	Yes
117	Ahmadieh	2019	RCT	Ahmadieh, H.; Nourinia, R.; Hafezi-Moghadam, A.; Sabbaghi, H.; Nakao, S.; Zandi, S.; Yaseri, M.; Tofighi, Z.; Akbarian, S. Intravitreal Injection of a Rho-Kinase Inhibitor (Fasudil) Combined with Bevacizumab versus Bevacizumab Monotherapy for Diabetic Macular Oedema: A Pilot Randomised Clinical Trial. *British Journal of Ophthalmology* **2019**, *103*, 922–927, doi:10.1136/bjophthalmol-2018-312244.	Yes	Yes						CT	Yes	Yes	Yes	Yes					
118	Minami	2019	Comparative retrospective study	Minami, Y.; Song, Y. seok; Ishibazawa, A.; Omae, T.; Ro-mase, T.; ishiko, S.; Yoshida, A. Effect of Ripasudil on Diabetic Macular Edema. *Scientific Reports* **2019**, *9*, 5–9, doi:10.1038/s41598-019-40194-5.	Yes	Yes											Yes	Yes	Yes	Yes	Yes

119	Glendenning	2018	Preclinical trial	Glendenning, A.; Crews, K.; Sturdivant, J.; A deLong, M.; Kopczynski, C.; Lin, C.-W. Sustained Release, Biodegradable PEA Implants for Intravitreal Delivery of the ROCK/PKC Inhibitor AR-13503 | IOVS | ARVO Journals. *Invest. Ophthalmol. Vis. Sci.* **2018**, *59*, 5672.	Yes	Yes	Yes	Yes	Yes	Yes	Yes
120	Ding	2019	Preclinical trial	Ding, J.; Crews, K.; Carbajal, K.; Weksler, M.; Moore, L.; Carlson, E.C.; Lin, C.-W. Ocular Tissue Distribution and Duration of Release of AR-13503 Following Administration of AR-13503 Sustained Release Intravitreal Implant in Rabbits and Miniature Swine | IOVS | ARVO Journals. *Invest. Ophthalmol. Vis. Sci.* **2019**, *60*, 5387.	Yes	Yes	Yes	Yes	Yes	Yes	Yes

RCT: Randomized Controlled Trial; CT: Clinical Trial
